# Blood Pressure Control among Adults with Hypertension at a Tertiary Hospital in Ethiopia

**DOI:** 10.4314/ejhs.v33i4.2

**Published:** 2023-07

**Authors:** Binyam Barega, Lissane Seifu, Addisu Melkie, Sintayehu Abebe, Melaku Taye

**Affiliations:** 1 Department of Internal Medicine, Ras Desta Hospital, Addis Ababa, Ethiopia; 2 Department of Internal Medicine, College of Health Sciences, Addis Ababa University, Addis Ababa, Ethiopia

**Keywords:** Hypertension, blood pressure control, tertiary hospital, Ethiopia

## Abstract

**Background:**

Uncontrolled hypertension is a leading modifiable risk factor for cardiovascular disease morbidity and mortality. Despite the availability of several effective blood pressure lowering drugs, hypertension control rates remain poor globally. This study aimed to define the level of blood pressure control and to determine the factors associated with poor hypertension control.

**Methods:**

A hospital-based cross-sectional study was conducted from January to March 2019 at Tikur Anbessa Specialized Hospital among randomly selected 369 patients with hypertension. Data were collected using a pre-tested structured questionnaire. Multivariate binary logistic regression was used to identify determinants of blood pressure control.

**Results:**

The mean (SD) age of the study participants was 55.5 (13.2) years; 188 (50.9%) were males and 28 (7.6%) were active smokers. More than half of the patients (56.0%) were overweight or obese. The most commonly identified comorbidities were diabetes mellitus (48.0%), dyslipidemia (50.9%), and chronic kidney disease (56.1%). The mean (SD) systolic blood pressure was 140.6 (22) mmHg, and diastolic blood pressure was 85.8 (14) mmHg. About two-thirds of the patients (60.2%) had uncontrolled blood pressure. The factors associated with poor blood pressure control with an AOR (95% CI) were increasing age: 1.05 (1.00-1.11), increasing household income: 1.25 (1.04-1.49), being physically inactive: 7.64 (1.14-51.13), chronic kidney disease: 5.36 (1.14-5.16), and use of home blood pressure monitoring: 0.31 (0.102-0.94).

**Conclusion:**

The rate of blood pressure control in patients with hypertension was suboptimal. Age, household income, level of physical activity, chronic kidney disease, and use of home blood pressure monitoring were independent predictors of blood pressure control. It is important to optimize the treatment of hypertension in this high-risk group by implementing effective strategies.

## Introduction

Hypertension is a growing global public health challenge that affects more than one billion people. It is a major contributor to the global burden of cardiovascular morbidity and mortality. It is responsible for the majority of stroke and coronary heart disease problems. Almost three-quarters of people with hypertension live in developing countries with limited health resource (1, 2).

Despite the established benefits of treating hypertension to target with available pharmacological and non-pharmacological therapies, hypertension remains inadequately controlled in clinical practices (3, 4). In developed and developing countries, less than 27 and 10% of hypertensive patients, respectively, achieve the target blood pressure (BP) (5).

Ethiopia is one of the highly affected countries in sub-Saharan Africa. Systematic reviews of observational studies in Ethiopia have estimated the prevalence of hypertension to be 20-30% with a higher prevalence in urban populations (6, 7). Uncontrolled hypertension among adult hypertensive patients ranged from 37% in Gondar to 53% in Jimma (8, 9).

Despite the high prevalence and poor control of hypertension in developing countries, the prevalence and factors associated with poor control have not been examined extensively in Ethiopia. Therefore, this study assessed BP control and its determinants in Tikur Anbessa Specialized Hospital using a well-validated digital automatic device for the first time.

## Materials and Methods

**Study Area, design and period**: An institution-based cross-sectional study was conducted at the renal clinic in Tikur Anbessa Specialized Hospital (TASH), Addis Ababa, Ethiopia. This study was conducted between January and March 2019.

**Study population**: The study population included adults with hypertension who attended the renal clinic at TASH for regular follow-up during the study period.

**Sample size and sampling technique**: Epi info version 7 was used to calculate the sample size, which was determined to be 374 using the formula to estimate a single population proportion. A simple random sampling technique was used to select the study participants.

**Inclusion and exclusion criteria**: All adults with hypertension, with at least one prior visit to the clinic and who were taking pharmacological therapy, were included.

**Data collection tools and procedures**: Four trained nurses collected the data by interviewing patients using a standard questionnaire, reviewing their medical records, and taking physical measurements. Data on sociodemographic and behavioral factors were collected through face-to-face interviews using the “World Health Organization (WHO) STEPS Instrument for Chronic Disease Risk Factor Surveillance” (10).

To measure self-care practice, the hypertension self-care activities scale effect (H-Scale) was used, which included medication adherence, low-salt diet, physical activity, smoking, weight management, and alcohol consumption (11). Data on the clinical and laboratory profiles of the participants were obtained through a review of their digital records using a data extraction tool. Blood pressure and anthropometric data were collected by direct measurements. Blood pressure was measured with a well-validated OMRON-M6 digital BP apparatus, which is capable of recording multiple BP readings automatically, following the standard BP measurement procedure (12, 13).

**Operational definitions**: Hypertension was defined as an elevated average BP with Systolic BP (SBP) ≥ 140 mmHg and/or Diastolic BP (DBP) ≥ 90 mmHg) or taking antihypertensive medication. Controlled BP was measured as BP < 140/90 mmHg according to the Eighth Joint National Committee (JNC8) (14).

**Data quality assurance, processing and management**: The collected data were validated and exported to the Statistical Package for Social Sciences (SPSS) version 25 for analysis. Descriptive statistics included the mean with Standard Deviation (SD) and median with interquartile ratio (IQR) for continuous variables, while frequency and percentage tables were used for categorical data. Simple cross-tabulation and binary logistic regression analyses were used to study the association between independent variables and the rate of hypertension control. Model fitness was checked using the Hosmer-Lemeshow goodness-of-fit test and was found to be fit. First, a bivariate analysis was performed to identify the variables associated with BP control. Variables with a P-value <0.25 in the bivariable analysis were selected as candidate variables and entered together into multivariable analysis to control for confounders. Lastly, variables with a p-value <0.05 in a multivariable analysis were considered statistically significant, and the adjusted odds ratio (AOR) with 95% Confidence Interval (CI) was estimated to measure the strength of the associations. The results are presented in text and tables.

**Ethical considerations**: Ethical approval was obtained from the departmental review board of Internal Medicine, College of Health Sciences, Addis Ababa University. Written informed consent was obtained from all subjects. The data were analyzed anonymously.

## Results

**Demographic and clinical characteristics of the respondents**: A total of 369 patients participated in the study, yielding a response rate of 98.7%. One hundred eighty-eight participants (50.9%) were males. The mean age was 55.5 (13) years, and 42.4% of them were older than 60 years. Most participants (82.9%) were urban residents.

The mean duration of antihypertensive drug therapy was 8.6 (10.9) years. The mean body mass index (BMI) was 26.2 (5.20) kg/m^2^. The most commonly identified comorbidities were diabetes mellitus (48.0%), dyslipidemia (51.0%), and chronic kidney disease (56.1%) (Table 1).

**Table 1:** Socio-demographic and clinical profile of patients with hypertension on follow-up at Tikur Anbessa Specialized Hospital, Addis Ababa, Ethiopia, 2019

Variable (n = 369)	Category	Frequency	(%)
**Age (years)**	18 – 29	15	4.1
	30 – 39	28	7.6
	40 – 49	70	19.0
	50 – 59	99	26.9
	≥ 60	157	42.4
**Marital Status**	Single	45	12.2
	Married	247	66.9
	Divorced	25	6.8
	Widowed	52	14.1
**Educational Status**	No formal education	70	19.0
	Primary education	94	25.5
	Secondary education	82	22.2
	Higher education	123	33.3
**Occupation**	Government Employee	56	15.2
	NGO Employee	55	14.9
	Self employed	121	32.8
	Unemployed	137	37.1
**Average household income**	≤1000	44, 173	11.9, 46.9
**(Ethiopian Birr)**	1000 – 2000	48	13.0
	2000 – 3000	36	9.8
	≥3000	68	18.4
**BMI (kg/m^2^)**	<25	155, 10	42.0, 2.7
	25 – 29.9	135	36.6
	≥30	69	18.7
**Duration of hypertension**	<5	186	50.4
**(years)**	5-10	87	23.6
	>10	96	26
**Common comorbidities**	CKD	207	56.1
	Dyslipidemia	188	50.9
	Diabetes Mellitus	177	48.0

NGO = Non-Government Organization, BMI = Body Mass Index, CKD = Chronic Kidney Disease

The percentage of current smokers was 7.6%, while 8.1% of the patients chewed khat. The practice of a salt-restricted diet was very poor, with only 16.0% of patients reporting adherence to salt restriction, whereas 9.0% of patients did not adhere to their antihypertensive medications. Only 5.4% of patients reported performing regular physical exercise. One-third (32.0%) of the patients checked their BP at home with their own BP measuring devices (Table 2).

**Table 2:** Hypertension self-care practice in patients with hypertension in Tikur Anbessa Specialized Hospital, Addis Ababa, Ethiopia, 2019

Variable (n = 369)	Category	Frequency	%
Medication adherence	Adherent	336	91.0
	Not adherent	33	9.0
Salt restriction adherence	Adherent	59	16.0
	Not adherent	310	84.0
Cigarette smoking	Yes	28	7.6
	No	341	92.4
Khat use	Yes	30	8.1
	No	339	91.7
Alcohol use	Yes	192	43.9
	No	177	47.9
Physical activity	Physically active	20	5.4
	Physically inactive	349	94.6
Uses home blood pressure	Yes	118	32.0
monitoring	No	251	68.0

**Blood pressure control levels of the respondents**: The mean (SD) SBP was 140.6 (22) mmHg, while the mean (SD) DBP was 85.8 (14) mmHg. The rate of blood pressure control was 39.8%. Half of the participants (49.9%) were taking monotherapy (angiotensin-converting enzyme inhibitors (ACEI)/angiotensin receptor blockers (ARB) 27.1%, Calcium channel blockers (CCB) 17.3%, Hydrochlorothiazide (HCT) 3.3%, others 2.2%), one-third (34.1%) were taking dual therapy (ACEI/ARB + CCB 11.4%, CCB + HCT 4.6%, ACEI/ARB + HCT 4.1%, CCB + beta blockers (BB) 2.4%) and the rest were taking three or more antihypertensive drugs.

**Determinants of uncontrolled blood pressure levels**: The results of the multivariable analysis identified five variables as independent determinants of BP control. For each one-year increase in the age of a participant, the odds of having poor BP control increased by 5% (AOR = 1.05; 95% CI: 1.00 - 1.11). As income increases by 1000 Ethiopian Birr (ETB), the odds of having poor BP control increases by 24% (AOR = 1.25; 95% CI: 1.04 - 1.49). Besides, the odds of having poor BP control were seven times higher in patients with physical inactivity (AOR = 7.64, 95% CI: 1.14-51.13). Likewise, patients with Chronic Kidney Disease (CKD) had a five times higher likelihood of having poor BP control (AOR = 5.36; 95% CI: 1.14 - 5.16). The odds of having poor BP control in patients who use home BP monitoring practice was reduced by 70% (AOR = 0.31; 95% CI: 0.102 - 0.94). There was a trend, albeit not statistically significant, toward poorer BP control among overweight/obese hypertensive patients than among patients with a normal BMI (62.0% vs. 58.3%, P = 0.475). Other comorbidities, duration of hypertension, smoking, alcohol intake, and khat chewing were not significantly associated with the level of blood pressure control in this study (Table 3).

**Table 3:** Determinants of blood pressure control among patients with hypertension at Tikur Anbessa Specialized Hospital, Addis Ababa, Ethiopia, 2019

Explanatory variable		BP control		COR (95%, CI)	AOR (95%, CI)
		Controlled	Uncontrolled		
Age (mean, years)		55.2 (±13.2)	55.62 (±13.3)	1.00 (0.99-1.02)	1.05 (1.00-1.11)
Income (mean, ETB in thousands)		2.8 (±2.5)	3.73 (±3.6)	1.10 (0.99-1.22)	1.25 (1.04-1.49)
Sex	Male	78 (41.5%)	110 (58.5%)	1	1
	Female	69 (38.1%)	112 (61.9%)	1.15 (0.76-1.75)	2.71 (0.88-8.36)
Physical activity	Active	4 (20%)	16 (80%)	1	1
	Inactive	143 (41%)	206 (59%)	2.78 (0.91-8.48)	7.63 (1.14-51.13)
CKD	Yes	68 (32.9%)	139 (67.1%)	1.95 (1.27-2.97)	5.36 (1.14-25.16)
	No	79 (48.8%)	83 (51.2%)	1	1
Diabetes mellitus	Yes	62 (35%)	115 (65%)	1	1
	No	85 (44.3%)	107 (55.7%)	0.68 (0.45-1.03)	0.71 (0.25-2.02)
Use of home BP monitoring	Yes	50 (42.4%)	68 (57.6%)	0.86 (0.55-1.34)	0.31 (0.102-0.93)
	No	97 (38.6%)	154 (61.4%)	1	1

ETB = Ethiopian Birr, BP = Blood Pressure, CKD = Chronic Kidney Disease

## Discussion

This study showed that the overall control of BP in adults with hypertension at TASH was low with only 39.8% achieving BP control targets. These results are consistent with other hospital-based studies conducted in different parts of Ethiopia with a 36.4 - 50.3% BP control rate (9, 15-17). The proportion of BP control was higher in the national STEPS survey (53.4%) (18). Similarly, a low BP control rate was reported in Kenyan (33.4%), Cameroon (36.8%), and South African (42.0 %) studies (19-21). The small differences among these studies may be due to differences in baseline patient characteristics and variations in methodologies used.

Multivariate analysis revealed that increasing age, increasing household income, being physically inactive, comorbid chronic kidney disease, and not checking BP at home were significant predictors of poor blood pressure control.

Older age has been identified as an important risk factor for poor blood pressure control in several studies (21-23). BP becomes difficult to control as age increases due to worsening peripheral vascular resistance resulting from loss of elasticity of vasculature with aging.

CKD was associated with poor BP control. This finding also agrees with those studies in Portugal (24) and Korea (25), which showed lower rates of BP control in hypertensive patients with CKD. Compared to patients with diabetes mellitus, those without diabetes mellitus were 30% less likely to have poorly controlled BP, although the difference was not statistically significant. This finding disagrees with those of several other studies showing lower rates of well-controlled blood pressure in diabetic hypertensive patients (21, 26, 27). Patients who had diabetes might have been paid closer attention by their physicians, other healthcare givers, and their BP could have been kept relatively better.

The use of self BP monitoring practice at home resulted in better BP control in this study. A systematic review of several other studies supported similar effects of self-monitoring on a higher rate of BP control (28, 29). The use of home BP monitoring practice likely encourages patient-centered care reminding patients of the importance of adherence to a healthy lifestyle and therapy, thereby improving BP control.

Physical inactivity was associated with a higher rate of uncontrolled hypertension. Several studies have evidenced that performing physical activities have a strong and independent roles in reducing blood pressure (30-32).

In contrast, higher household income was associated with a lower likelihood of achieving BP control. This is discordant with many studies demonstrating that lower income and socioeconomic status are strongly associated with poor BP control due to lack of access to healthcare, lower knowledge of health behavior, and social support (33-35). It could be thought that those with higher income might be physically less active from using walking than transportation, or adopting a relatively more expensive western-type food, negatively affecting BP control.

The main limitations of this study were that it was a single-center study and the possibility of social desirability bias on self-reported behavioral and lifestyle factors. Although a well-validated OMRON-M6 digital automatic device was used for BP measurements, 24-hour ambulatory BP monitoring was not done to assess the white-coat effect and masked hypertension.

This study showed that the majority of hypertensive patients had poorly controlled blood pressure. Increasing age, increasing household income, being physically inactive, comorbid chronic kidney disease, and not using home BP monitoring were significantly associated with uncontrolled BP. Therefore, targeted interventions with these predictive factors are important for achieving high BP control and improving the care of patients with hypertension.
